# Asymptomatic Transmission of SARS-CoV-2 on Evacuation Flight

**DOI:** 10.3201/eid2611.203353

**Published:** 2020-11

**Authors:** Sung Hwan Bae, Heidi Shin, Ho-Young Koo, Seung Won Lee, Jee Myung Yang, Dong Keon Yon

**Affiliations:** Soonchunhyang University College of Medicine, Seoul, South Korea (S.H. Bae);; Soonchunhyang University Seoul Hospital, Seoul (S.H. Bae);; Harvard Business School, Boston, Massachusetts, USA (H. Shin);; Korea University College of Medicine, Seoul (H.-Y. Koo);; Sejong University College of Software Convergence, Seoul (S.W. Lee);; University of Ulsan College of Medicine, Seoul (J.M. Yang);; Asan Medical Center, Seoul (J.M. Yang);; Armed Force Medical Command, Republic of Korea Armed Forces, Seongnam, South Korea (D.K. Yon);; CHA University School of Medicine, Seongnam (D.K. Yon)

**Keywords:** COVID-19, SARS-CoV-2, severe acute respiratory syndrome coronavirus 2, viruses, respiratory infections, zoonoses, coronavirus disease, aircraft, asymptomatic transmission, Italy, South Korea

## Abstract

We conducted a cohort study in a controlled environment to measure asymptomatic transmission of severe acute respiratory syndrome coronavirus 2 on a flight from Italy to South Korea. Our results suggest that stringent global regulations are necessary for the prevention of transmission of this virus on aircraft.

Undocumented cases of severe acute respiratory syndrome coronavirus 2 (SARS-CoV-2) infection have been common during the coronavirus disease (COVID-19) global pandemic (*1**–**3*). Although inflight transmission of symptomatic COVID-19 has been well established (*1*,*2*), the evidence for transmission of asymptomatic COVID-19 on an aircraft is inconclusive. We conducted a cohort study evaluating asymptomatic passengers on a flight that carried 6 asymptomatic patients with confirmed SARS-CoV-2 infections. The Institutional Review Board of Armed Force Medical Command approved the study protocol. The ethics commission waived written informed consent because of the urgent need to collect data on COVID-19.

## The Study

On March 31, 2020, we enrolled in our study 310 passengers who boarded an evacuation flight from Milan, Italy, to South Korea. This evacuation flight was conducted under strict infection control procedures by the Korea Centers for Disease Control and Prevention (KCDC), based on the guidelines of the World Health Organization (WHO) (*4*). When the passengers arrived at the Milan airport, medical staff performed physical examinations, medical interviews, and body temperature checks outside the airport before boarding, and 11 symptomatic passengers were removed from the flight. Medical staff dispatched from KCDC were trained in infection control under the guidance of the KCDC and complied with the COVID-19 infection protocol, which was based on WHO guidelines (*4*). N95 respirators were provided, and passengers were kept 2 m apart for physical distancing during preboarding. Most passengers wore the N95 respirators except at mealtimes and when using the toilet during the flight. After an 11-hour flight, 299 asymptomatic passengers arrived in South Korea and were immediately quarantined for 2 weeks at a government quarantine facility in which the passengers were completely isolated from one another. Medical staff examined them twice daily for elevated body temperature and symptoms of COVID-19. All passengers were tested for SARS-CoV-2 by reverse transcription PCR twice, on quarantine day 1 (April 2) and quarantine day 14 (April 15). 

Asymptomatic patients were those who were asymptomatic when they tested positive and did not develop symptoms within 14 days after testing (*5*). Among the 299 passengers (median age 30.0 years; 44.1% male), 6 had a confirmed positive result for SARS-CoV-2 on quarantine day 1 and were transferred immediately to the hospital (Table). At 14 days after the positive test, the 6 patients reported no symptoms and were categorized as asymptomatic.

**Table:** Baseline characteristics and quarantine day 1 SARS-CoV-2 test results for asymptomatic passengers from flight from Milan, Italy, to South Korea, March 2020*

Characteristics	All asymptomatic passengers, N = 299	Passengers testing negative for SARS-CoV-2, n = 293	Patients testing positive for SARS-CoV-2, n = 6
Median age (IQR), y	30.0 (27.0–35.0)	30.0 (27.0–35.0)	28.0 (9.9–45.0)
Sex			
M	132 (44.1)	128 (43.7)	4 (66.7)
F	167 (55.9)	165 (56.3)	2 (33.3)
Underlying conditions			
Diabetes	1 (0.3)	1 (0.3)	0 (0.0)
Hypertension	6 (2.0)	6 (2.0)	0 (0.0)
Asthma	1 (0.3)	1 (0.3)	0 (0.0)
Coronary artery disease	1 (0.3)	1 (0.3)	0 (0.0)
Cancer	3 (1.0)	3 (1.0)	0 (0.0)
Connective tissue disease	1 (0.3)	1 (0.3)	0 (0.0)
Liver disease	1 (0.3)	1 (0.3)	0 (0.0)
Thyroid disease	2 (0.7)	2 (0.7)	0 (0.0)
Current pregnancy	4 (1.4)	4 (1.4)	0 (0.0)
Charlson Comorbidity Index score			
0	287 (96.0)	281 (95.9)	6 (100.0)
1	8 (2.7)	8 (2.7)	0 (0.0)
>2	4 (1.3)	4 (1.4)	0 (0.0)

*Values are no. (%) except as indicated. IQR, interquartile range; SARS-CoV-2, severe acute respiratory syndrome coronavirus 2.

On quarantine day 14, a 28-year-old woman who had no underlying disease had a confirmed positive test result for COVID-19. On the flight from Milan, Italy, to South Korea, she wore an N95 mask, except when she used a toilet. The toilet was shared by passengers sitting nearby, including an asymptomatic patient. She was seated 3 rows away from the asymptomatic patient (Figure). Given that she did not go outside and had self-quarantined for 3 weeks alone at her home in Italy before the flight and did not use public transportation to get to the airport, it is highly likely that her infection was transmitted in the flight via indirect contact with an asymptomatic patient. She reported coughing, rhinorrhea, and myalgia on quarantine day 8 and was transferred to a hospital on quarantine day 14. The remaining 292 passengers were released from quarantine on day 15.

**Figure:**
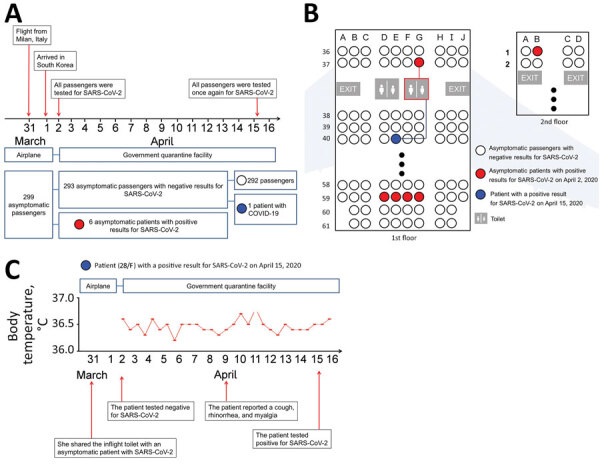
Cohort study of severe acute respiratory syndrome coronavirus 2 (SARS-CoV-2) transmission on evacuation flight from Milan, Italy, to Incheon, South Korea, on March 31, 2020. Red indicates asymptomatic patients who tested positive on quarantine day 1 (April 1, 2020); blue indicates asymptomatic patient who tested positive on quarantine day 14 (April 15, 2020). A) Timeline of flight, passenger quarantine, and testing protocol. B) Location of 6 asymptomatic patients and subsequently infected patient on flight. C) Case report of patient apparently infected during evacuation flight.

All crew members (n = 10) and medical staff dispatched from KCDC (n = 8) were quarantined at a government quarantine facility for 2 weeks and were tested twice for SARS-CoV-2, on quarantine days 1 and 14. All 18 members of the cabin crew and medical staff were negative for SARS-CoV-2 on both occasions.

To reinforce our results, we performed an external validation using a different dataset. Another evacuation flight of 205 passengers from Milan, Italy, to South Korea on April 3, 2020, was also conducted by KCDC under strict infection control procedures. Among the passengers on this flight were 3 asymptomatic patients who tested positive on quarantine day 1 and 1 patient who tested negative on quarantine day 1 and positive on quarantine day 14. On the basis of an epidemiologic investigation, the authors and KCDC suspect that this infection was also transmitted by inflight contact.

## Conclusions

This study was one of the earliest to assess asymptomatic transmission of COVID-19 on an aircraft. Previous studies of inflight transmission of other respiratory infectious diseases, such as influenza and severe acute respiratory syndrome, revealed that sitting near a person with a respiratory infectious disease is a major risk factor for transmission (*6*,*7*), similar to our own findings. Considering the difficulty of airborne infection transmission inflight because of high-efficiency particulate-arresting filters used in aircraft ventilation systems, contact with contaminated surfaces or infected persons when boarding, moving, or disembarking from the aircraft may play a critical role in inflight transmission of infectious diseases (*6*,*7*).

Previous studies reported that viral shedding can begin before the appearance of COVID-19 symptoms (*8*,*9*), and evidence of transmission from presymptomatic and asymptomatic persons has been reported in epidemiologic studies of SARS-CoV-2 (*5*,*10*,*11*). Because KCDC performed strong infection control procedures during boarding; the medical staff and crew members were trained in infection control; all passengers, medical staff, and crew members were tested twice for SARS-CoV-2; and a precise epidemiologic investigation was conducted, the most plausible explanation for the transmission of SARS-CoV-2 to a passenger on the aircraft is that she became infected by an asymptomatic but infected passenger while using an onboard toilet. Other, less likely, explanations for the transmission are previous SARS-CoV-2 exposure, longer incubation period, and other unevaluated situations. 

The control measures incorporated into our cohort study provide a higher level of evidence than previous studies on asymptomatic transmission (*5*,*10*,*11*). Our findings suggest the following strategies for the prevention of SARS-CoV-2 transmission on an aircraft. First, masks should be worn during the flight. Second, because contact with contaminated surfaces increases the risk for transmission of SARS-CoV-2 among passengers, hand hygiene is necessary to prevent infections. Third, physical distance should be maintained before boarding and after disembarking from the aircraft. 

Our research provides evidence of asymptomatic transmission of COVID-19 on an airplane. Further attention is warranted to reduce the transmission of COVID-19 on aircraft. Our results suggest that stringent global regulations for the prevention of COVID-19 transmission on aircraft can prevent public health emergencies.
